# Enhancing Natural Killer Cell-Mediated Cancer Immunotherapy by the Biological Macromolecule *Nocardia rubra* Cell-Wall Skeleton

**DOI:** 10.3389/pore.2022.1610555

**Published:** 2022-08-30

**Authors:** Jie Wu, Baojun He, Miao Miao, Xibin Han, Hongyan Dai, Heng Dou, Yanqiu Li, Xiaoqing Zhang, Guangchuan Wang

**Affiliations:** ^1^ Department of Oncology, The First Affiliated Hospital of Jinzhou Medical University, Jinzhou, China; ^2^ Department of Clinical Laboratory Medicine, The First Affiliated Hospital of Jinzhou Medical University, Jinzhou, China; ^3^ Department of Immunology, School of Basic Medical Science, Jinzhou Medical University, Jinzhou, China; ^4^ Laboratory Animal Center, Jinzhou Medical University, Jinzhou, China; ^5^ Department of Outpatient PICC, The First Affiliated Hospital of Jinzhou Medical University, Jinzhou, China; ^6^ Greatest Biopharma Limited Company, Benxi, China; ^7^ Teaching Center for Basic Medical Experiment, China Medical University, Shenyang, China

**Keywords:** cancer, metastasis, natural killer cell, *Nocardia* rubra cell-wall skeleton, cytotoxicity

## Abstract

The biological macromolecule *Nocardia rubra* cell-wall skeleton (Nr-CWS) has well-established immune-stimulating and anti-tumor activities. However, the role of Nr-CWS on natural killer (NK) cells remains unclear. Here, we explore the function and related mechanisms of Nr-CWS on NK cells. Using a tumor-bearing model, we show that Nr-CWS has slightly effect on solid tumor. In addition, using a tumor metastasis model, we show that Nr-CWS suppresses the lung metastasis induced by B16F10 melanoma cells in mice, which indicates that Nr-CWS may up-regulate the function of NK cells. Further investigation demonstrated that Nr-CWS can increase the expression of TRAIL and FasL on spleen NK cells from Nr-CWS treated B16F10 tumor metastasis mice. The spleen index and serum levels of TNF-α, IFN-γ, and IL-2 in B16F10 tumor metastasis mice treated with Nr-CWS were significantly increased. *In vitro*, the studies using purified or sorted NK cells revealed that Nr-CWS increases the expression of CD69, TRAIL, and FasL, decreases the expression of CD27, and enhances NK cell cytotoxicity. The intracellular expression of IFN-γ, TNF-α, perforin (prf), granzyme-B (GrzB), and secreted TNF-α, IFN-γ, IL-6 of the cultured NK cells were significantly increased after treatment with Nr-CWS. Overall, the findings indicate that Nr-CWS could suppress the lung metastasis induced by B16F10 melanoma cells, which may be exerted through its effect on NK cells by promoting NK cell terminal differentiation (CD27^low^CD11b^high^), and up-regulating the production of cytokines and cytotoxic molecules.

## Introduction

*Nocardia rubra* is a species of gram-positive, rod-shaped bacteria that is not pathogenic to human [1]. *Nocardia rubra* cell-wall skeleton (Nr-CWS) was first reported in 1976 [2]. The compositional analysis revealed that Nr-CWS is composed of 0.35% D-mannose, 0.63% D-ribose, 2.60% L-rhamnose monohydrate, 1.83% D-ructose, 5.92% D-glucose, 2.54% D-xylose, 32.36% D-lactose, and 53.76% arabinose. Early investigations have reported that Nr-CWS is with high immuno-stimulating and anti-tumor activities, and could directly stimulate macrophages, CD4^+^, and CD8^+^ T cells [2-7]. In combination with chemotherapy or through administration locally at the tumor site, Nr-CWS prolongs the survival of patients with advanced cancers and increase the levels of cytokines, such as IL-1, IL-6, TNF-α and IFN, in tumor tissue [8]. In addition, Nr-CWS can improve the therapeutic efficacy of PD-1/PD-L1 immunosuppressive pathway in patients with high-risk human papillomavirus (HR-HPV) infection and cervical precancerous lesion, cervical intraepithelial neoplasia (CIN), and mouse CT26. WT cancer cell [6, 9]. Our previous study showed that Nr-CWS can promote CD4^+^ T cells activation, drive Th1 immune response, and enhance CD8^+^ T cells function [4, 5]. Recently, the application of Nr-CWS products has been approved in China (approval Number S20030009), for cervical cancer treatment. However, whether Nr-CWS is able to modulate the function of NK cells in tumor metastasis is still under elucidation.

NK cells are major players of innate immunity. They respond quickly against various types of pathogens [10] and play critical roles in immuno-surveillance against tumors [11]. It has been well established that NK cells participate in many physiological and pathophysiological processes, including immuno-surveillance against tumors, anti-intracellular pathogen infection, allograft rejection, and pregnancy [12, 13]. NK cells can remove targets through different pathways [14], including the release of cytotoxic molecules (like perforin, granzymes), induction of apoptosis through TRAIL and FasL, and directly killing targets through antibody-dependent cellular cytotoxicity (ADCC) [15]. NK cells are functionally heterogeneous, which are divided into different subgroups of maturation with different levels of cytokines and cytotoxic capacity based on the expression of CD27 and CD11b [16]. Accumulated evidence shows that NK cells can acquire function through a broad spectrum of molecular and cellular mechanisms [11, 15–17]. In this study, using *in vitro* and *in vivo* models, we found that Nr-CWS could suppress the lung metastasis of B16F10 melanoma model, which may be through its effect on NK cells by promoting NK cell terminal differentiation (CD27^low^CD11b^high^) and up-regulating the production of cytokines and cytotoxic molecules.

## Materials and Methods

### Reagents

Nr-CWS was supplied by Greatest Biopharma Limited Company (purity>98%). Fluorescein conjugated anti-mouse antibodies APC-NK1.1, FITC-NK1.1, APC/Cy7-CD3ε, PE/Cy7-CD3ε, APC-CD4, PE-CD8α, PerCP/Cy5.5-CD11b, PE-CD27, FITC-TNF-α, and PerCP/Cy5.5-IFN-γ from BioLegend, and PerCP/Cy5.5-CD69, FITC-FasL, PE-TRAIL, PE-Perforin, and FITC-Granzyme-B from eBioscience were used in this study. TUNEL assay kit and MTS Cell proliferation, cytotoxicity or chemosensitivity assays kit (cat # 3580) were obtained from Promega. The LEGENDplex™ Multiplex mouse cytokine kit was provided by BioLegend. EasySep™ mouse NK cell isolation kit was from STEMCELL Technologies Inc. Recombinant murine IL-2 was from PeproTech Inc.

### Tumor Models and *Nocardia rubra* Cell-Wall Skeleton Administration

Female C57BL/6 mice (6–8 weeks, 18–20 g) supplied by Beijing Vital River Laboratory Animal Technology, were maintained in specific pathogen-free animal rooms and treated following the guideline for Care and Use of Laboratory Animals of Jinzhou Medical University. Lewis lung carcinoma (LLC) and B16F10 melanoma cell lines were bought from Cell Bank of Chinese Academy of Sciences in Shanghai. The cells were grown in DMEM medium supplemented with 10% (v/v) fetal bovine serum (FBS), 100 U/ml penicillin and 100 μg/ml streptomycin, in a humidified atmosphere of 5% CO_2_ at 37°C. LLC cells (5×10^6^/ml in PBS) were subcutaneously injected in the medial axillas of 22 C57BL/6 mice (0.1 ml/mouse) and after establishment of tumor model for 24 h (hrs), mice were divided into two groups randomly (11 mice/group, Nr-CWS group and control group). For experimental metastasis, B16F10 melanoma cells (1×10^6^/ml in PBS) were injected into C57BL/6 mice through tail-vein (0.2 ml/mouse) and the injected mice were divided into two groups randomly 24 h later (4 mice/group, Nr-CWS group, control group). Nr-CWS group were given Nr-CWS by intra-peritoneal injection (10 mg/kg in PBS, once-daily) for 7 or 14 successive days, and the control group were treated with equivalent PBS.

### Sample Processing

For the mouse tumor-bearing model (11 mice/group), 5 mice from each group were euthanized with an overdose of isoflurane on day 7 after tumor cell inoculation. Then solid tumors were dissected for analyses of tumor weight and the percentage of tumor-infiltrating lymphocytes. After 14 days, the remained mice (6 mice/group) were euthanized for sampling: the solid tumors were dissected, each tumor sample was sectioned for tumor morphology and tumor cell apoptosis assay. For experimental metastasis model (one mice in control group died during experiment was excluded), pulmonary metastases were assessed by comparing the numbers of metastases on day 14 after tumor cell injection. Blood serum and spleen were obtained from the metastasis model mice. Index for spleen was calculated as previously reported [18].

### Analysis of Tumor Morphology by Hematoxylin and Eosin Staining and Tumor Cells Apoptosis by TUNEL

Tumor morphology was analyzed as previously described [19]: in brief, tumor tissues from Nr-CWS treated and control groups (day 14) were fixed using paraformaldehyde, Fixed samples were embedded in wax after dehydration and clearing using a series of ethanol solution and xylene, thin slices (4 μm) were cut and mounted on microscope slides, samples were stained using hematoxylin and eosin stain (H&E) after deparaffinized and rehydration. Finally slides were mounted using mounting medium and analyzed [19].

Tumor cell apoptosis was detected using TUNEL Assay Kit, the ratio of positive cells per specimen was analyzed under a light microscope. For each sample, five most obvious positive staining areas were analyzed [20]. TUNEL positive cells were counted and apoptotic index (AI) was calculated in reference of a previous publication [20].

### Analysis of Tumor Infiltrating Lymphocytes

Each tumor sample dissected on day 7 after tumor cell inoculation was digested using trypsin (0.05%) and mashed through moisturized cell strainer (70 μm), then the single-cell suspension was collected and counted. 1×10^6^ cells were taken for staining. The cocktail of antibodies (APC-CD4, PE/Cy7-CD3ε, FITC-NK1.1, and PE-CD8α) was added into the cell suspension and then the stained samples were incubated for 20 min at 4°C. For dead cell exclusion, 5 µl of 7-Aminoactinomycin D (7-AAD) was used for each sample before analysis. Then samples were detected using flow cytometry (FCM) (BD FACS Canto II) and 30,000 events were acquired and analyzed using BD Diva software.

### Natural Killer Cell Purification and Cell Culture

Spleens from wild type C57BL/6 mice were mashed through a moisturized cell strainer. NK cell was purified with EasySep™ mouse NK cell isolation kit and its purity was evaluated by FCM. For the cytokines analysis in culture supernatant and NK cell cytotoxicity assay, purified NK cells were subjected to fluorescence-activated cell sorting (BD FACSAriaIII) to exclude other cell contamination using CD3ε and NK1.1 staining. Freshly isolated NK cells or sorted NK cells were seeded in 96-well flat-bottom plate (1×10^5^/well) containing RPMI-1640 complete medium supplemented with 10% FBS, 100 U/ml penicillin and 100 μg/ml streptomycin, 50 μM β-mercaptoethanol, 500 U/ml IL-2, and treated with or without 15 μg/ml Nr-CWS (the optimal dose for splenocytes from our previous experiments) in a humidified atmosphere of 5% CO_2_ at 37°C. After 24 or 48 h incubation respectively, NK cells were collected for staining. The culture supernatant was collected for multi-cytokines analysis by FCM (BD Canto II).

### Analysis of Cell Surface Molecules Expression by Flow Cytometry

For NK cells analysis from *in vivo* experiment, spleen single-cell suspension were made by mashing whole spleen through a moisturized cell strainer and RBC lysing. For cultured NK cells analysis *in vitro*, NK cell was purified from spleen single-cell suspension with EasySep™ mouse NK cell isolation kit. NK cells from spleen of Nr-CWS treated and control B16F10 metastasis mice (after 14 days treatment), and the cultured wild type (WT) spleen NK cells incubated with or without Nr-CWS (15 μg/ml) at indicated time-points were collected for analysis of surface molecules expression by FCM. And cells from each sample were suspended in 100 μl staining buffer. A cocktail of antibodies (combination of the fluorescent conjugated antibodies CD3ε, NK1.1, TRAIL, FasL, CD69 or CD3ε, NK1.1, CD11b, and CD27) was used and the stained samples were incubated for 20 min (4°C). After washing with PBS, the stained cells were analyzed by FCM.

### Analysis of Intracellular Perforin/Granzyme-B and Cytokines

Purified WT NK cells treated with or without Nr-CWS (15 μg/ml) were collected at indicated time-points. Monensin (BioLegend) was present for the last 5 h before collection. Then, samples were stained with APC-NK1.1 and APC/Cy7-CD3ε antibodies for surface markers. For intracellular staining, samples were fixed and permeabilized after surface staining using Fixation Buffer (cat # 420801) and Intracellular Staining Permeabilization Wash Buffer (10×, cat # 421002). Then, samples were further stained with the cocktail of cytotoxic molecules (PE-Perforin, FITC-Granzyme B) or cytokines (FITC-TNF-α, PerCP/Cy5.5-IFN-γ) antibodies. After intracellular staining and extensive washing, samples were subjected to FCM analysis.

### Multiplex Analysis of Cytokines by Flow Cytometry

The cytokines in the mouse serum of metastasis model and WT NK cell culture supernatants were measured using the LEGENDplex™ Multiplex mouse cytokine kit from BioLegend by FCM as we have reported [5]. The serum from Nr-CWS treated (for 14 days) metastasis mice and control mice were collected and stored at −80°C. The supernatants of cultured NK cells treated with or without Nr-CWS (15 μg/ml) were collected at indicated time-points. Cytokines (IL-2, IL-4, IL-5, IL-6, IL-10, IFN-γ and TNF-α) were evaluated in all serum and supernatant samples. Results were analyzed using LEGENDplex software as previously reported [4, 5].

### MTS Assay for Natural Killer Cell Cytotoxicity *In Vitro*

The assay for NK cell cytotoxicity was performed as previously described by MTS assay [19]. Briefly, NK cells sorted from wild type C57BL/6 mice were stimulated with or without Nr-CWS (15 μg/ml) for 24 h and collected as effectors after washing. LLC cells were serviced as targets. The effector NK cells were mixed with LLC cells (5×10^3^/well) at effector:target ratios of 10:1 and 5:1, and incubated in 96-well flat-bottom plate (Corning-Costar) in 100 µl volume for 6 h. At the last 1 hour, 20 µl/well of CellTiter 96^®^ AQueous One Solution Reagent (MTS) was added. After 1 h at 37°C in a humidifi ed, 5% CO_2_ atmosphere, the optical density (OD) was measured using multiskan spectrum microplate spectrophotometer at 490 nm from triple wells. The cytotoxicity was calculated as previous report [19]:

$$\text{Cytolytic activity}\left(\text{\%}\right)=\{1-\frac{\text{OD of }\left(\text{target cells }+\text{ NK cells}\right)-\text{OD of NK cells}}{\text{OD of target cells}}\}\times 100$$

### Statistical Analysis

Data were shown using mean ± standard deviation (SD). The Independent-Samples t Test was used to analyze results in SPSS 16.0 (SPSS, Inc., Chicago, IL, United States). The *p*-value <0.05 was considered as statistically significant, as shown in the figure legends. Data shown in figures are representative of three independent experiments.

## Results

### The Weight and Histopathology of Solid Tumors

**FIGURE 1:**
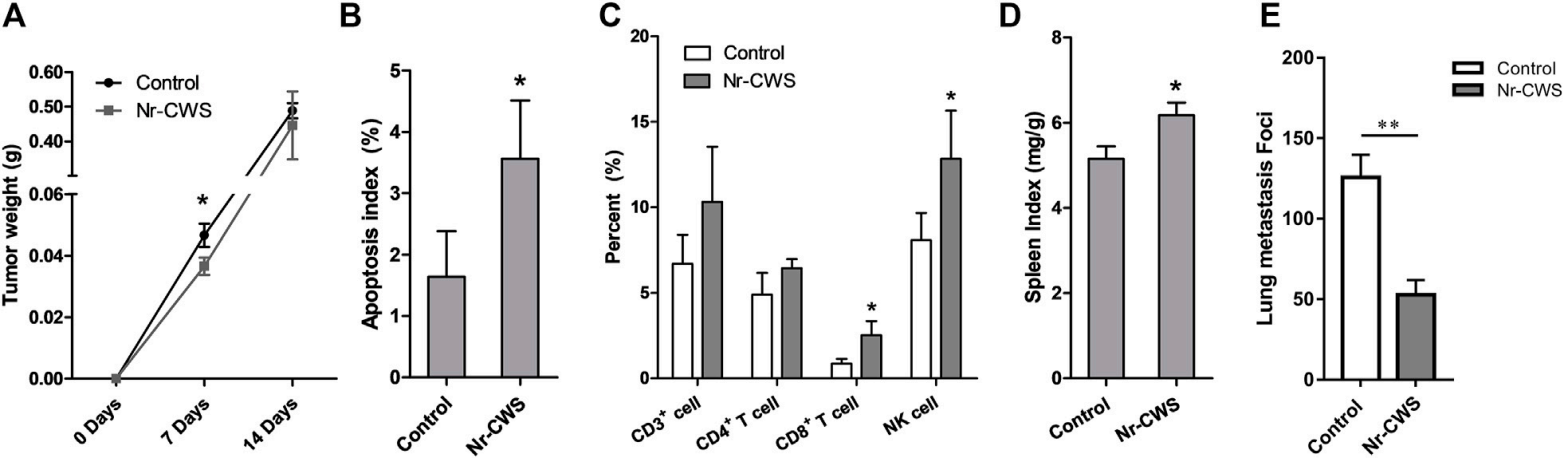
Potential enhancement of anti-tumor immune responses by Nr-CWS. **(A)** The statistical analysis of tumor weights of Nr-CWS treated mice compared with that of control mice at day 7 and 14. **(B)** The analysis of apoptotic cells in tumor tissues by TUNEL. Histogram showing the apoptotic index of two study groups. **(C)** Tumor infiltrating lymphocytes were analyzed by FCM. The histogram showed the percentage of CD3^+^, CD4^+^ T, CD8^+^ T, and NK cells in Nr-CWS treated mice and control mice. **(D)** The spleen index of Nr-CWS treated mice and control mice (after 14 days treatment). **(E)** Quantification of metastases from mice treated with or without Nr-CWS at day 14 after tail vein injection of B16F10 melanoma cells (0.2 M/mouse). Data are shown as means ± SD; *, *p* < 0.05; **, *p* < 0.01.

To confirm the anti-tumor activity of Nr-CWS, C57BL/6 wild type mice were implanted with LLC cells and then treated with either Nr-CWS or PBS control. The results showed that the average tumor weight of the Nr-CWS group was decreased after 7 days treatment when compared to the control group (*p* < 0.05). However, it was comparable after 14 days treatment (*p* > 0.05) (Figure 1A; Supplementary Figure S1A). The histopathological examination of tumors in the Nr-CWS group (after 14 days treatment) showed larger necrotic areas and more prominent infiltration of inflammatory cells than that in the control group. H&E staining showed the tumor cells were poorly differentiated and heterogeneous, with hyperchromatic nuclei and scant cytoplasm. There were also binucleated or multinucleated cells in the tumor samples. The representative histopathological changes are shown in Supplementary Figures S1B,C.

### Analysis of Tumor Cells Apoptosis by TUNEL

The TUNEL System was used for detecting apoptotic cells and apoptotic bodies *in situ* of solid tumors. The morphological changes of apoptotic cells were observed as follows: apoptotic DNA fragments were stained brown mainly in the condensed nuclei (Supplementary Figure S1D). The apoptotic index was calculated using the percentage of stained brown cells at ×400 magnification. Results showed that Nr-CWS treatment caused more tumor cell apoptosis and higher apoptotic index compared with the control group (*p* < 0.05, Figure 1B, Supplementary Figure S1D).

### Analysis of Tumor Infiltrating Lymphocytes, Spleen Index, and B16F10 Lung Metastasis

For each tumor sample from the tumor-bearing mice after 7 days treatment with or without Nr-CWS, the percentage of infiltrated lymphocytes including CD3^+^, CD4^+^ T, CD8^+^ T, and NK cells, were analyzed by FCM (Figure 1C, Supplementary Figure S1E). The percentage of CD8^+^ T and NK cells were significantly increased after Nr-CWS treatment (*p* < 0.05), while the percentage of CD3^+^ and CD4^+^ T cells were only slightly up-regulated (*p* > 0.05) (Figure 1C, Supplementary Figure S1E).

The spleen index of the Nr-CWS treated group was significantly increased after 14 days treatment (*p* < 0.05), while the spleen index of the Nr-CWS and control group did not show any significant difference (data not shown) after 7 days treatment, as shown in Figure 1D.

To further investigate the role of Nr-CWS *in vivo*, we adopted the B16F10 melanoma metastasis model. Results showed Nr-CWS treatment can significantly reduce the lung metastasis compared to the control group in Figure 1E, Supplementary Figure S1F (*p* < 0.01).

### Analysis of Natural Killer Cell Purity by Flow Cytometry

The NK cells were purified using EasySep™ mouse NK cell isolation kit based on Magnetic-activated cell sorting (MACS). All living cells (7-AAD negative) were analyzed. The percentage of NK cell in spleen before purification was 2.63 ± 0.31%. After purification, the percentage of NK cell was >80% (81.97 ± 3.40%) as determined by FCM gating on NK1.1^+^ CD3ε^−^ population (Supplementary Figure S1G). For the cytokines analysis in culture supernatant and NK cell cytotoxicity assay, purified NK cells were subjected to fluorescence-activated cell sorting to exclude other cell contamination using CD3ε and NK1.1 staining. The purity of sorted NK cell was more than 98% (data not shown).

### Analysis the Expression of Natural Killer Cell Functional Markers

**FIGURE 2:**
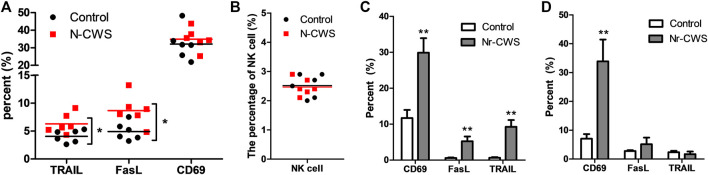
Expression of functional molecules of NK cells after Nr-CWS treatment *in vivo* and *in vitro*. The graphs showed the percentage of CD69, TRAIL, and FasL positive NK cells *in vivo* [**(A)**, *n* = 6] and *in vitro* [**(C)**, 24 h; **(D)**, 48 h], and the percentage of spleen NK cells in Nr-CWS treated mice and control mice [**(B)**, *n* = 6]. Data are shown as means ± SD; *, *p* < 0.05; **, *p* < 0.01.

To assess the effect of Nr-CWS on the functional molecules of NK cells, FCM analysis was performed to determine the expression of functional markers on NK cells gated the population of NK1.1^+^ CD3ε^−^ cells. The *in vivo* samples showed that the expression of TRAIL and FasL on splenic NK cell were much higher in the Nr-CWS group than the control group (*p* < 0.05, Figure 2A, Supplementary Figure S2A). However, CD69 expression and splenic NK cell percentage were comparable between the two groups (*p* > 0.05, Figures 2A,B). Results of the *in vitro* experiment (Supplementary Figures S2B,C) showed Nr-CWS can strikingly increase the expression of CD69, TRAIL, and FasL on NK cells after incubation for 24 h (*p* < 0.01, Figure 2C) and increase CD69 expression after incubation for 48 h (*p* < 0.01, Figure 2D).

### Analysis of Perforin/Granzyme-B Expression *In Vitro*

**FIGURE 3:**
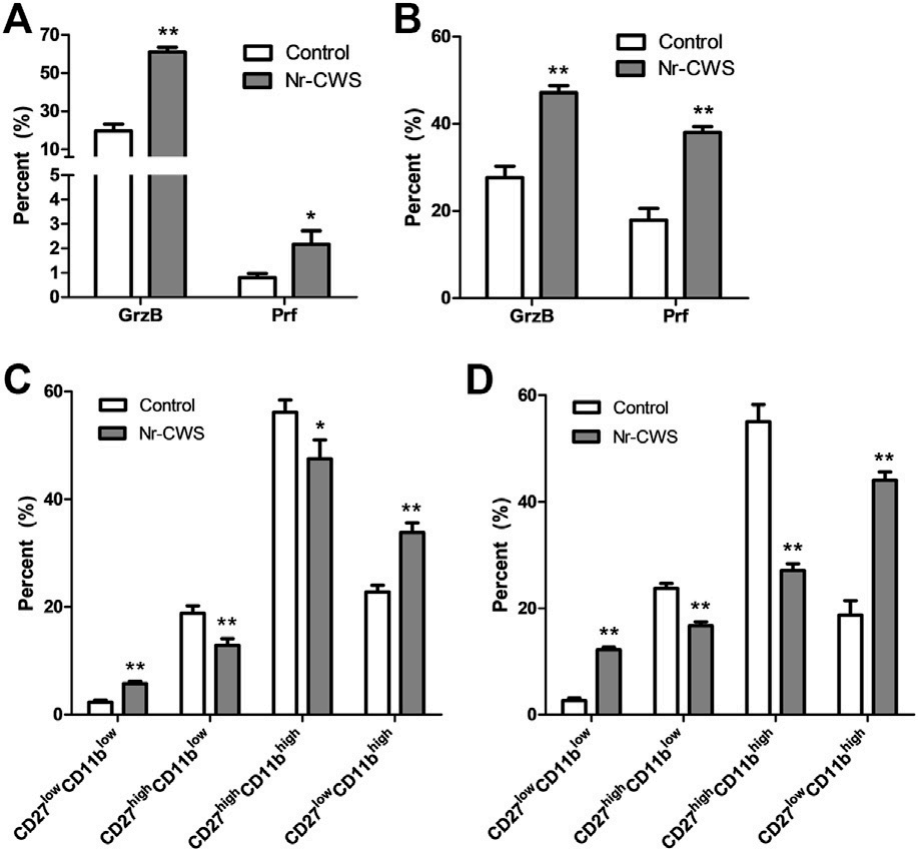
Analysis of NK cell subgroups and the expression of perforin and granzyme B *in vitro*. **(A,B)** The statistical analyses of the percentage of Prf and GrzB of the Nr-CWS treated and control NK cells by flow cytometry [**(A)**, 24 h; **B**, 48 h]. **(C,D)** The statistical analyses of NK cell subgroups based on the expression of CD11b and CD27 by flow cytometry. Graphs showed the statistical analyses of the Nr-CWS treated and control NK cells [**(C)**, 24 h; **(D)**, 48 h]. Data are shown as means ± SD; *, *p* < 0.05; **, *p* < 0.01.

To investigate the effect of Nr-CWS on NK cell cytotoxicity, the expression of intracellular perforin and granzyme-B of NK cells (gated on NK1.1^+^ CD3ε^−^) treated with or without Nr-CWS for 24 or 48 h, were analyzed by FCM. Results showed that Nr-CWS significantly increased the expression of granzyme-B and perforin after incubation for 24 and 48 h, as shown in Figures 3A,B, Supplementary Figures S3A,B.

### Analysis of Natural Killer Cell Subgroups *In Vitro*

Purified normal spleen NK cells treated with or without Nr-CWS were collected at indicated time-points for subgroups analysis by FCM gating on NK1.1^+^CD3ε^−^ population. Our data showed that Nr-CWS treatment can significantly up-regulate the percentage of CD27^low^CD11b^high^ and CD27^low^CD11b^low^ cells, and down-regulate the percentage of CD27^high^CD11b^low^ and CD27^high^CD11b^high^ cells after incubation for 24 and 48 h, as shown in Figures 3C,D and Supplementary Figures S3C,D.

### Analysis of Intracellular Cytokines

**FIGURE 4:**
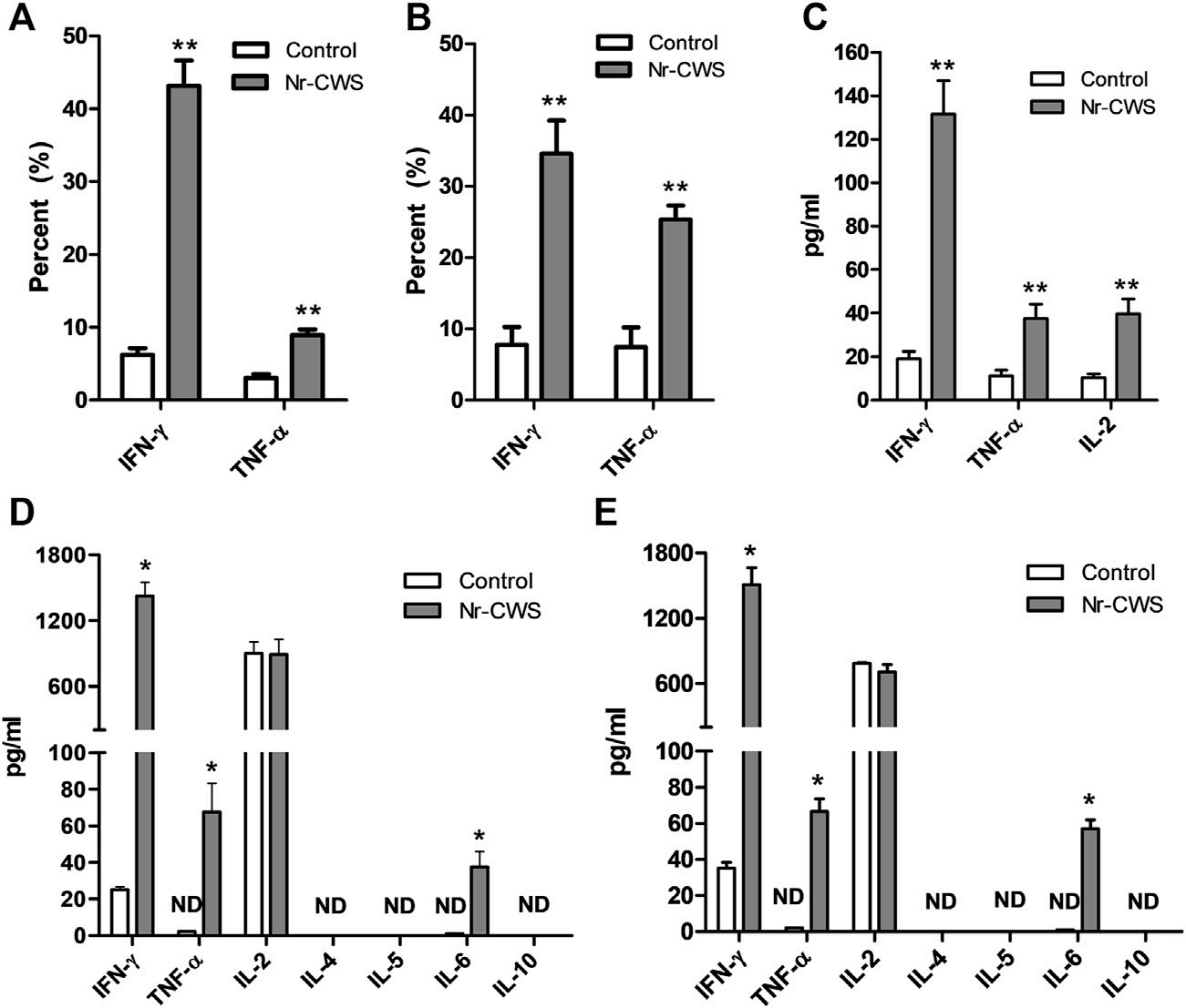
Analysis of cytokines. **(A,B)** Quantification of the expression of IFN-γ and TNF-α of NK cells treated with or without Nr-CWS by flow cytometry. The statistical analyses of the expression of IFN-γ and TNF-α of NK cells treated with or without Nr-CWS *in vitro* [**(A)**, 24 h; **(B)**, 48 h]. **(C)** The statistical analyses of the cytokines (IFN-γ, TNF-α, IL-2) in serum after Nr-CWS treatment *in vivo*. **(D,E)** The statistical analyses of secreted cytokines (IL-2, IL-4, IL-5, IL-6, IL-10, TNF-α, and IFN-γ) in the supernatants of cultured NK cells treated with or without Nr-CWS *in vitro* [**(D)**, 24 h; **(E)**, 48 h]. Data are shown as means ± SD; ND, not detected; *, *p* < 0.05; **, *p* < 0.01.

Purified normal spleen NK cells treated with or without Nr-CWS were collected at indicated time-points for intracellular cytokine analysis by FCM gating on NK1.1^+^CD3ε^−^ population. Results showed that Nr-CWS can stimulate NK cells to produce significantly more IFN-γ and TNF-α after treatment for 24 and 48 h (*p* < 0.01, Figures 4A,B, Supplementary Figures S4A,B).

### Analysis of Secreted Cytokines in Serum and Natural Killer Cell Culture Supernatant

The levels of cytokines in the supernatant and serum from NK cell culture and animals treated with or without Nr-CWS were analyzed by FCM using LEGENDplex™. The serum levels of IL-2, IFN-γ, and TNF-α were significantly higher after Nr-CWS treatment *in vivo* (*p* < 0.01, Figure 4C). Results of cytokines in the cultured supernatants showed Nr-CWS significantly increased the production of IFN-γ and TNF-α after treatment for 24 and 48 h *in vitro*. In contrast, the level of IL-2 was comparable between the two groups (*p* > 0.05). Nr-CWS can drive the expression of IL-6, which were 37.51 ± 8.32 and 57.16 ± 4.80 pg/ml after incubation for 24 and 48 h *in vitro*, whereas it was not detectable (ND) in the control group, as shown in Figures 4D,E.

### Natural Killer Cells Mediated Cytotoxicity *In Vitro*

**FIGURE 5:**
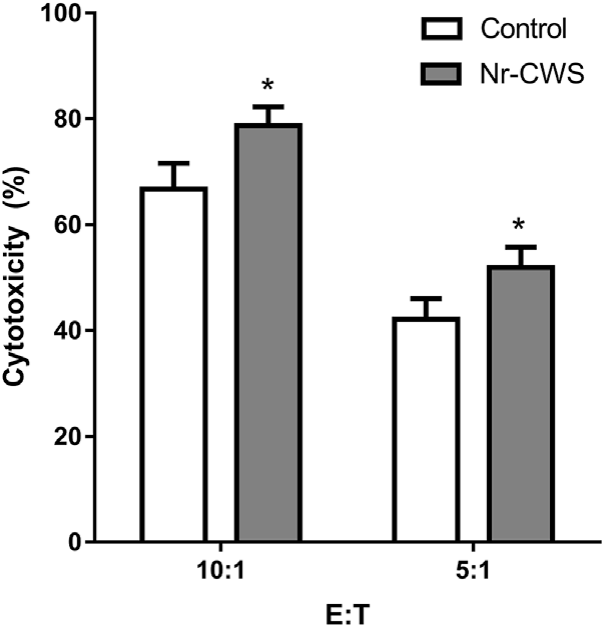
Cytotoxicity of NK cells treated with Nr-CWS against LLC cells. Histogram showed the statistical analysis of NK cell cytotoxicity in the Nr-CWS group versus that in the control group at the indicated E:T ratios. Data shown are representative of at least three independent experiments, and calculated data are shown as means ± SD; *, *p* < 0.05.

To measure the cytotoxic activity, sorted NK cells were treated with or without Nr-CWS for 24 h. Then the NK cells were washed and the number was accounted, and mixed with LLC cells at indicated ratios for 6 h. The cytotoxic activity was measured by MTS assay. Results showed that Nr-CWS can strikingly increase NK cell-mediated cytotoxicity at both effector:target ratios of 10:1 and 5:1 (*p* < 0.05, Figure 5).

## Discussion

NK cells, belonging to the innate immune system, unlike T and B cells, play essential roles in the anti-virus or anti-cancer responses by directly killing target cells through rapidly releasing cytotoxic molecules and cytokines, or transduction of apoptosis signaling using surface receptors without prior sensitization [21]. NK cells are efficient and timely to maintain immune homeostasis, especially in early stage of virus-infection and tumor development. In consideration of the compositions and immune-stimulatory function of Nr-CWS we and other groups have reported earlier [3-7], we further explored the immune-regulatory role of Nr-CWS on NK cell against cancer.

In our study, we first explored the anti-tumor effects of Nr-CWS using the LLC tumor-bearing C57BL/6 mice model *in vivo*. The results showed that there is no apparent effect of Nr-CWS on solid tumor and there is no significant difference of tumor weight at the late stage of tumor growth after Nr-CWS treatment, although Nr-CWS can slightly suppress the tumor growth at the early stage (treated for 7 days; Figure 1A, Supplementary Figure S1A) and induces tumor cell apoptosis (treated for 14 days; Figure 1B, Supplementary Figures S1B,D). It is interesting that the Nr-CWS treated tumor-bearing mice showed significantly up-regulated percentages of tumor infiltrating CD8^+^ T and NK cells at the early stage (treated for 7 days; Figure 1C, Supplementary Figure S1E), and higher spleen index (treated for 14 days; Figure 1D). A previous study indicated that NK cells can inhibit tumor growth once they enter a tumor site [22] and strikingly delay tumor formation in a model of spontaneous mammary carcinoma at the earliest stages of carcinogenesis rather than late stages in a perforin-dependent manner [23, 24]. Our findings suggest that Nr-CWS may prominently enhance NK cell function. To test this possibility, the B16F10 melanoma metastasis model, which is the most commonly used metastatic melanoma model for preclinical studies was adopted to evaluate the effect of Nr-CWS *in vivo* [25]. As expected, our result showed that Nr-CWS significantly reduced the B16F10 melanoma lung metastasis (Figure 1E, Supplementary Figure S1F), which may be exerted through its effect on NK cells.

To further understand the functional mechanisms behind Nr-CWS induced NK cell-mediated anti-tumor responses, the functional markers of NK cell playing essential roles in immuno-regulation, antiviral activity, and immune-surveillance were analyzed. As well known, activated NK cells express certain markers like CD69 [26] and functional molecules such as TRAIL and FasL, via which NK cells kill targets through apoptotic pathways [27, 28]. Our data indicates that Nr-CWS increased the pro-apoptotic molecules TRAIL and FasL expression of NK cells *in vivo* (Figure 2A, Supplementary Figure S2A).

To further explore related mechanisms, the purified or sorted NK cells treated with Nr-CWS were assessed by determining the expression of phenotype markers and cytotoxic molecules, production of intracellular and secreted cytokines, and cytotoxicity against tumor cells. The data revealed that the CD69, TRAIL, and FasL expression were strikingly up-regulated after 24 h treatment with Nr-CWS (Figures 2C,D, Supplementary Figures S2B,C), which is consistent with *in vivo* results. In addition to TRAIL and FasL, NK cells may exert their cytotoxic function directly through GrzB and Prf, which can perforate cell membranes and directly kill target cells [29]. Consistently, the suitable amount of Nr-CWS can significantly increase the production of GrzB and Prf of NK cells after 24 and 48 h cell culture (Figures 3A,B, Supplementary Figures S3A,B). NK cells were defined as CD27^low^CD11b^low^, CD27^high^CD11b^low^, CD27^high^CD11b^high^, and CD27^low^CD11b^high^ four subgroups by CD27 and CD11b expression. The CD27^high^ NK cell subgroups display the highest secretion of cytokines, while the CD27^low^CD11b^high^ subgroup is the terminally differentiated cells that have the strongest cytotoxic capacity [30–32]. Our data indicated that Nr-CWS decreased CD27 expression after treatment for 24 or 48 h (Figures 3C,D, Supplementary Figures S3C,D), suggesting that Nr-CWS could enhance NK cell function through promoting NK cell into CD27^low^CD11b^high^ terminally differentiated subgroup.

Besides their direct effect on target, NK cells, particularly the terminally differentiated ones, can regulate anti-tumor or anti-pathogen responses through secretion of different cytokines, chemokines, and growth factors [14, 33, 34], which have been related to NK cell terminal differentiation [35]. In order to get deeper insight into Nr-CWS induced NK cells maturation and cytokine production, we measured the levels of cytokines in the serum and supernatants. In both serum and the supernatants, the levels of IFN-γ and TNF-α were strikingly up-regulated after Nr-CWS treatment (Figure 4, Supplementary Figure S4), suggesting that Nr-CWS could enhance anti-tumor activities of NK cells through regulating cytokines production.

Finally, we also tested the cytotoxicity *in vitro* using sorted NK cells. The result indicated that Nr-CWS could augment cytotoxic activity of NK cells (Figure 5). In light of the cytokine analysis data (Figures 4D,E), it is reasonable to believe that Nr-CWS may enhance the anti-tumor activity and augmentation of NK cell functions. Taken together, our study revealed a new profile of NK cells activation by Nr-CWS and provided a new mode of action of Nr-CWS as a new immune modulator in cancer therapy. Although we understand that Nr-CWS promotes NK cell function and maturation, it still needs to study its underlying mechanisms. In future experiments, in order to elucidate its underlying mechanism of action, it may be necessary to identify Nr-CWS-binding receptors on NK cells, as well as intracellular signaling pathways.

## Conclusion

In conclusion, our study demonstrated that Nr-CWS could suppress the lung metastasis of B16F10 melanoma model, which may be through its effect on NK cells. The possible mechanisms include: up-regulation of the expression of FasL and TRAIL, promotion of the production of cytotoxic cytokines (IFN-γ, TNF-α) and granules (Prf, GrzB), promotion of NK cell differentiation into the terminally mature*d* CD27^low^ CD11b^high^ subgroup and enhancement of the cytotoxic activity of NK cell.

## Data Availability

The original contributions presented in the study are included in the article/Supplementary Material, further inquiries can be directed to the corresponding authors.
