# Supplementary material for: Enhancing Natural Killer Cell-Mediated Cancer Immunotherapy by the Biological Macromolecule Nocardia rubra Cell-Wall Skeleton
Source: Pathol Oncol Res. 2022 Aug 30;28:1610555. doi: 10.3389/pore.2022.1610555 (PMC9468226; doi:10.3389/pore.2022.1610555)
Supplement: Supplementary file 1 [file Presentation1.PPTX]

## Slide 1
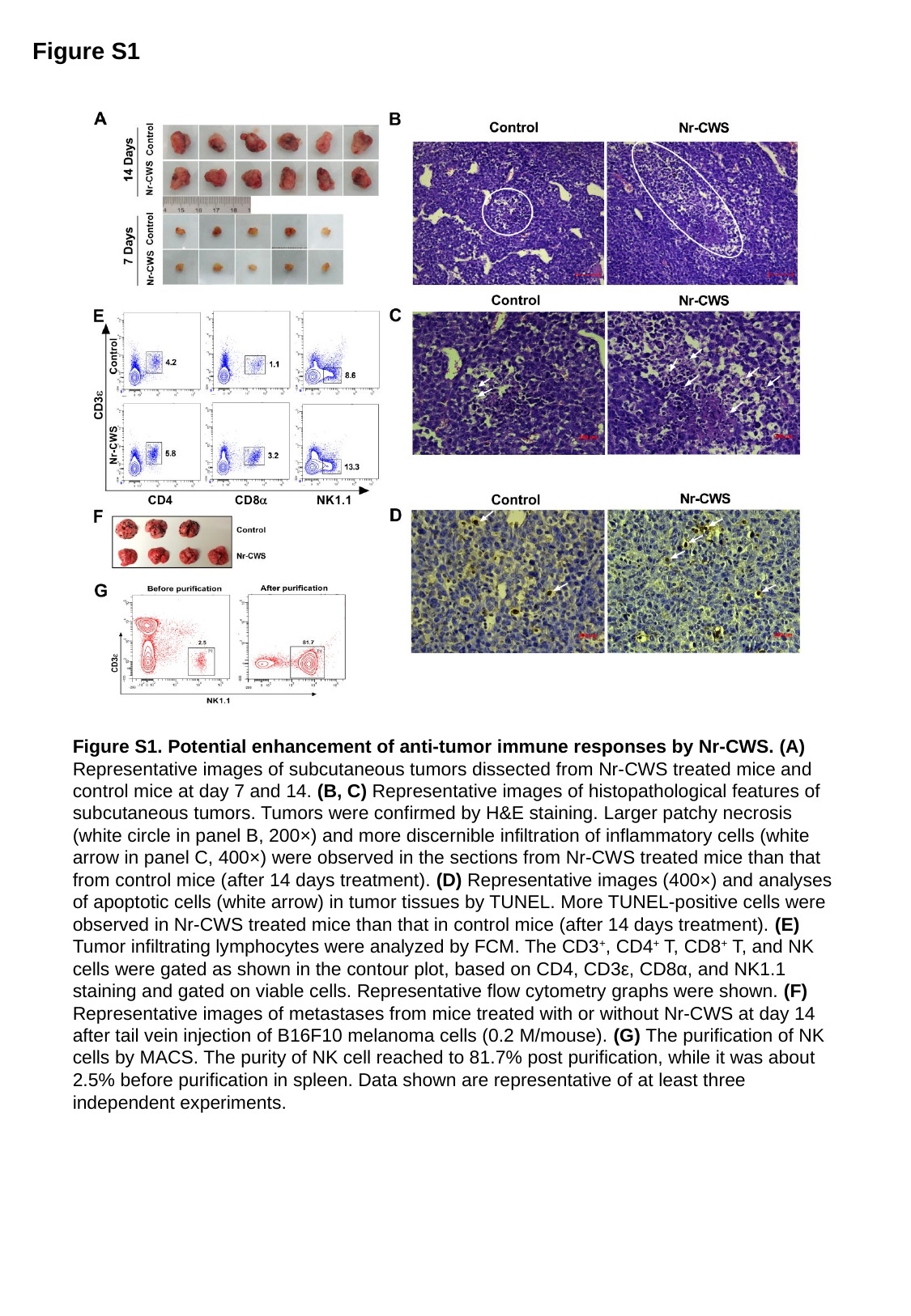

Figure S1
Figure S1. Potential enhancement of anti-tumor immune responses by Nr-CWS. (A) Representative images of subcutaneous tumors dissected from Nr-CWS treated mice and control mice at day 7 and 14. (B, C) Representative images of histopathological features of subcutaneous tumors. Tumors were confirmed by H&E staining. Larger patchy necrosis (white circle in panel B, 200×) and more discernible infiltration of inflammatory cells (white arrow in panel C, 400×) were observed in the sections from Nr-CWS treated mice than that from control mice (after 14 days treatment). (D) Representative images (400×) and analyses of apoptotic cells (white arrow) in tumor tissues by TUNEL. More TUNEL-positive cells were observed in Nr-CWS treated mice than that in control mice (after 14 days treatment). (E) Tumor infiltrating lymphocytes were analyzed by FCM. The CD3+, CD4+ T, CD8+ T, and NK cells were gated as shown in the contour plot, based on CD4, CD3ε, CD8α, and NK1.1 staining and gated on viable cells. Representative flow cytometry graphs were shown. (F) Representative images of metastases from mice treated with or without Nr-CWS at day 14 after tail vein injection of B16F10 melanoma cells (0.2 M/mouse). (G) The purification of NK cells by MACS. The purity of NK cell reached to 81.7% post purification, while it was about 2.5% before purification in spleen. Data shown are representative of at least three independent experiments.

## Slide 2
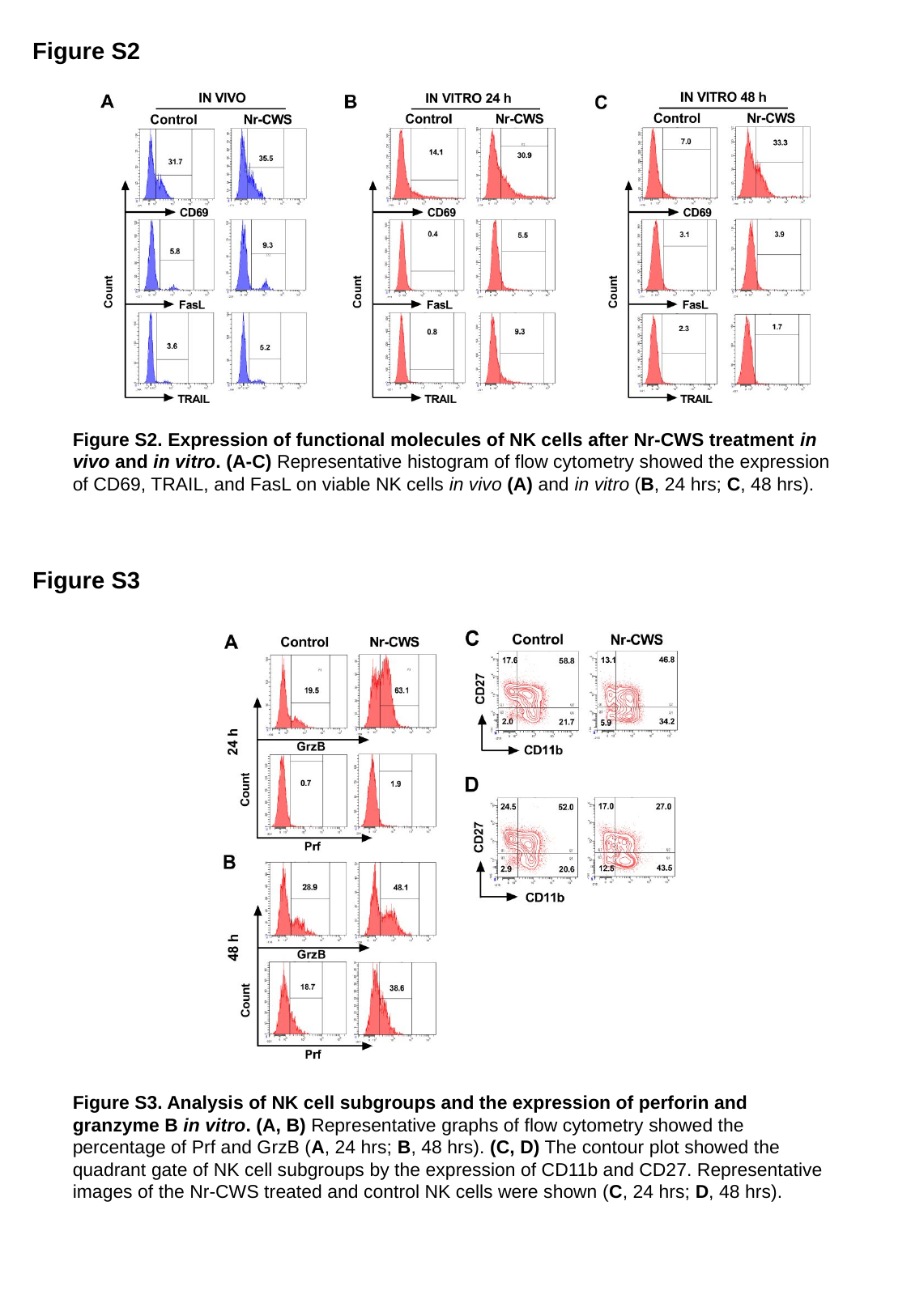

Figure S2
Figure S2. Expression of functional molecules of NK cells after Nr-CWS treatment in vivo and in vitro. (A-C) Representative histogram of flow cytometry showed the expression of CD69, TRAIL, and FasL on viable NK cells in vivo (A) and in vitro (B, 24 hrs; C, 48 hrs).
Figure S3
Figure S3. Analysis of NK cell subgroups and the expression of perforin and granzyme B in vitro. (A, B) Representative graphs of flow cytometry showed the percentage of Prf and GrzB (A, 24 hrs; B, 48 hrs). (C, D) The contour plot showed the quadrant gate of NK cell subgroups by the expression of CD11b and CD27. Representative images of the Nr-CWS treated and control NK cells were shown (C, 24 hrs; D, 48 hrs).

## Slide 3
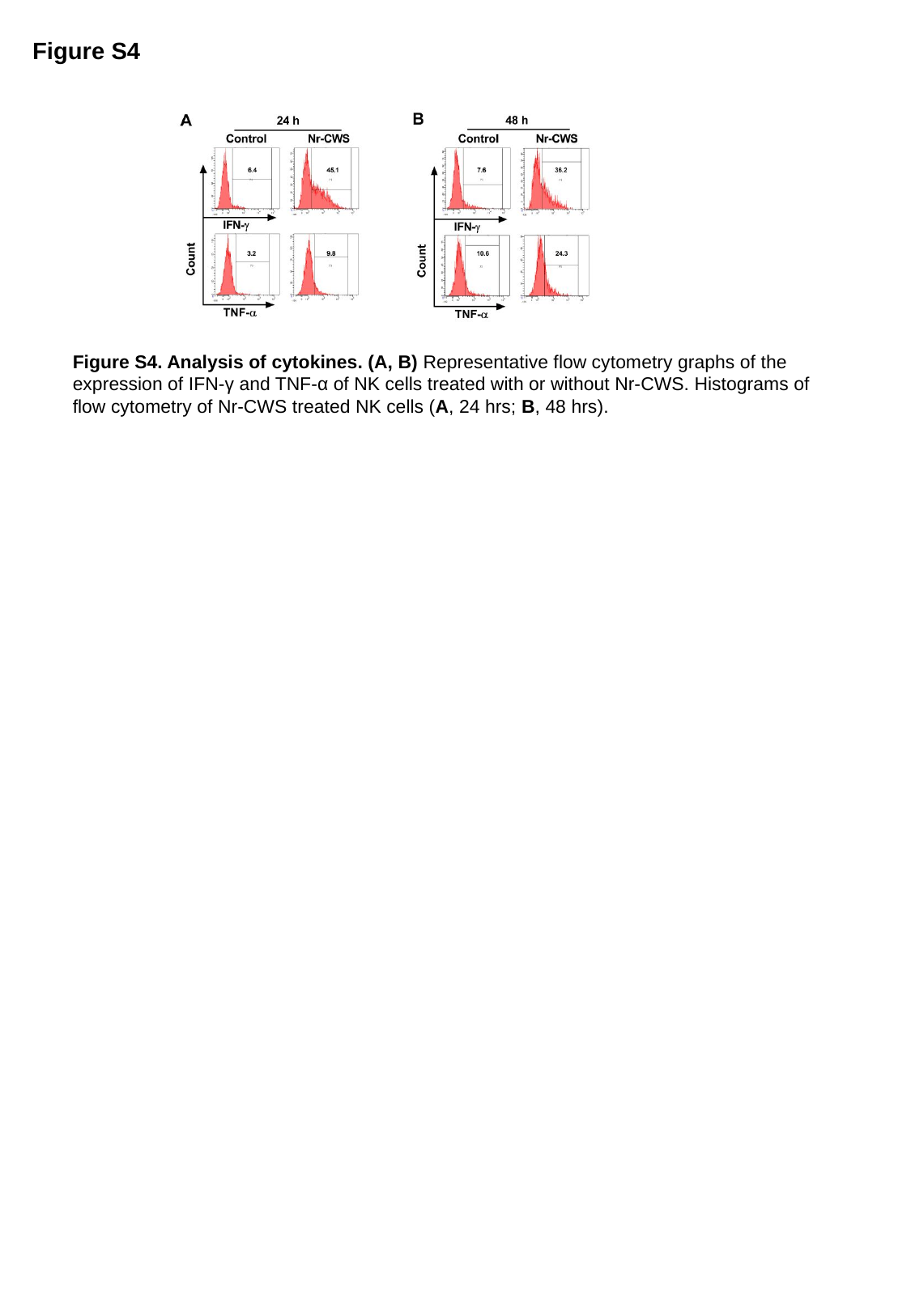

Figure S4
Figure S4. Analysis of cytokines. (A, B) Representative flow cytometry graphs of the expression of IFN-γ and TNF-α of NK cells treated with or without Nr-CWS. Histograms of flow cytometry of Nr-CWS treated NK cells (A, 24 hrs; B, 48 hrs).
